# Population Dynamics of Aphids on Cereals: Digging in the Time-Series Data to Reveal Population Regulation Caused by Temperature

**DOI:** 10.1371/journal.pone.0106228

**Published:** 2014-09-03

**Authors:** Marek Brabec, Alois Honěk, Stano Pekár, Zdenka Martinková

**Affiliations:** 1 Department of Nonlinear Modeling, Institute of Computer Science of the Academy of Sciences of the Czech Republic, Prague, Bohemia, Czech Republic; 2 Department of Invertebrate and Plant Biodiversity in Agrosystems, Crop Research Institute, Prague, Bohemia, Czech Republic; 3 Department of Botany and Zoology, Faculty of Science of the Masaryk University, Brno, Moravia, Czech Republic; University College Dublin, Ireland

## Abstract

Aphid populations show periodic fluctuations and many causes are attributed to their dynamic. We investigated the regulation by temperature of the aphid populations composed of *Metopolophium dirhodum*, *Sitobion avenae*, and *Rhopalosiphum padi* on winter wheat using a 24 years long time series data. We computed the sum of daily temperatures above 5°C, the threshold temperature for aphid development, and the sum of daily temperatures within the [0(threshold for wheat development),5] °C interval. Applying Generalised Additive Model framework we tested influences of temperature history expressed via degree days before the start of the aphid immigration on the length of their occurrence. We aimed to estimate the magnitude and direction of this influence, and how far to the past before the start of the aphid season the temperature effect goes and then identify processes responsible for the effect. We fitted four models that differed in the way of correcting for abundance in the previous year and in specification of temperature effects. Abundance in the previous year did not affect the length of period of aphid population growth on wheat. The temperature effect on the period length increased up to 123 days before the start of the current season, i.e. when wheat completed vernalization. Increased sum of daily temperatures above 5°C and the sum of daily temperatures within the [0,5] °C interval both shortened the length of period of aphid population growth. Stronger effect of the latter suggests that wheat can escape from aphid attacks if during winter temperatures range from 0 to 5°C. The temperature influence was not homogeneous in time. The strongest effect of past temperature was about 50 to 80 and 90 to 110 days before the beginning of the current aphid season indicating important role of termination of aphid egg dormancy and egg hatching.

## Introduction

Population size of organisms fluctuates in time and space due to variety of reasons, such as fluctuations in environmental conditions, availability of resources and impact of enemies. Changes in population numbers are variable but some general patterns have been revealed: large fluctuations occur in animals that occupy larger areas [1], have higher reproductive potential [2], are of smaller size [3] or are trophically specialized [4].

Aphids are economically significant pests attacking variety of crops. A considerable annual variation in aphid population numbers has been frequently observed. For example, in a previous study on winter wheat aphids in the central Czech Republic [5], the peak abundance varied during an 18-year period by two orders of magnitude. The aphids on cereals form a multispecies complex [6]–[8]. Such parallel occurrence of several aphid species is determined by similar life history: the species are holocyclic though overwintering on different primary hosts [9]. Dominant species are *Metopolophium dirhodum* (Walker) and *Rhopalosiphum padi* (L.), both heteroecic with eggs overwintering on roses (*Rosa* spp.) and bird-cherry (*Prunus padus* L.) respectively, and *Sitobion avenae* (F.), monoecic with eggs overwintering on grasses.

The seasonal fluctuation in their dynamic is determined by population growth rate and length of the period for which the population can grow. After passing few generations on the primary host in the spring, migration to cereal stands occurs in mid May at the cereal development stage of stem extension (Zadoks scale 31–32). Parthenogenetic females have high reproductive potential due to short generation time caused by “telescopic” mode of reproduction [10], which results in quick succession of generations and a high intrinsic rate of population increase. Aphid abundances grow quickly and may attain more than 100 individuals per tiller in late June or early July [11]. The peak is followed by an abrupt decline caused by host plant senescence and spreading of mycoses [12]. Aphid abundance plotted over time has thus a left-skewed triangular shape. The growth rate of aphid populations is affected by host plant quality (species and cultivars) and environmental conditions, particularly soil fertility and crop stand density resulting in intraspecific competition. Predators and parasitoids limit population growth as the population peak approaches [13].

Different factors were advocated in search for explaining cyclic dynamic of aphids: long-term trends in agriculture practices [5], weather changes [14], natural enemy abundance, and intraspecific competition [15]. Establishing causes of annual changes in aphid populations is of high interest and practical importance to farmers.

Here we focused on a hypothesis that annual variation of aphid populations is driven by temperature. Their maximum abundance is determined largely by duration of the period of aphid population growth. The onset of this period in the spring is determined by aphid migration to cereal stands, and the end of the period in summer is terminated by host plant maturation. Both processes are temperature driven but determined by different thermal constants. Lower thermal threshold for aphids was established by a number of authors who indicated the lower threshold to be between 0–6°C [16]–[20]. It is higher than that of the winter wheat which is virtually 0°C at early stage of development [21]. At later developmental stages the threshold for wheat may be higher and vary between cultivars [22]. Winter and early spring temperatures thus determine stage when aphids arrive on wheat crop and the length of period available to them before the onset of plant senescence. Winter temperatures between 0–5°C allow wheat development, postpone development of crop at which aphids arrive, and consequently shorten period of aphid population growth and decrease their maximum abundance. But if winter temperatures are less than 0°C, crop development is retarded and after the onset of higher temperature aphid populations have longer period available for their development and consequently can reach higher densities because the duration of period of aphid propagation is positively correlated with their maximum abundances [11]. Furthermore, aphid population size may be influenced by their numbers in the previous year as direct density-dependence of annual variation in aphid numbers was observed in some species [23], [24].

To test the hypothesis of temperature regulation we used data collected over 24 years and applied structured statistical models (i) to detect possible influences of temperature history before the start of the aphid immigration on the length of aphid population growth, and (ii) to estimate the magnitude and direction of this influence, and its temporal extent, i.e. how far to the past before the start of the aphid population growth the temperature effect goes. Since one can argue that the length of aphid population growth can be influenced also by the previous year's aphid density, we adjusted for the possible influence of internal aphid dynamics over the years by effectively separating the temperature-related and density-dependence variables.

## Materials and Methods

### Data

Aphid counts were made from 1987 through 2010 in production stands of winter wheat in Praha-Ruzyn (50°05′N, 14°10′E, 340 m a.s.l.) in the western Czech Republic. Specific permission for field work was not required and no endangered or protected species were involved. The cereals were cultivated following recommended agriculture practices [5]. No insecticides were applied during the study, and fungicides and liquid fertilizers were sprayed early in the season before aphids arrived and thus did not influence their population development. Every year, 3–6 sampling plots of 5×5 m size were established in a line transect across two adjacent wheat fields located at the Crop Production Institute in Praha-Ruzyn. Within a field plots were 30–50 m apart with a 30 m minimum distance from the field margin. The number of plots varied (3–15) but in most years there were six replicates. Aphid presence in the plots was monitored since the beginning of May, before aphid arrival to crops and ceased after aphid populations disappeared because of crop senescence, in July. For each year, date and plot, abundance of aphids was calculated as a sum of individuals of all three species on all tillers investigated. Each year, weekly aphid counts were conducted each date on 50 to 300 tillers per plot during 6–10 weeks of their presence on the wheat. For each date and plot, we then computed the density ( = total abundance found/total number of tillers investigated). We obtained weekly density time series for each plot when aphids were present in the wheat fields under investigations. The length of time series varied from year to year. As the number of tillers varied among sampling dates, the number was included in the variance function of the error term of all models. Data on aphid presence on cereals are available at http://www.vurv.cz/sites/file/cerealaphids.xls.

In the site of our study, alike in the western Czech Republic, aphid populations consist of three species, exclusively leaf populating *M. dirhodum* which usually is the most abundant species, and ubiquitous *S. avenae* and *R. padi*. Although they colonize different host plant organs the abundances are correlated on spatial as well as temporal scale.

Temperature, measured over the study period, was recorded at a meteorology station of the Crop Research Institute (http://www.vurv.cz/meteo/). Maximum distance of experimental plots from this station was <1 km. The temperature was recorded in 15 min intervals using a thermometer (range −50 to +110°C) placed at 2 m above ground surface. Daily temperature means used in this paper were calculated from these data.

Since, in this paper, we are interested in how temperature influences the time before the onset of the aphids on crops and the time when maximum density is reached, we have to derive a relevant characteristics. Namely, we computed time-from-start-to-maximum (TSM) as the number of days between the time of the first non-zero density and the time when the observed density reached its maximum, for each year and plot separately.

The influence of temperature is based on the traditional concept of degree days (DD). This means that, effectively, the calendar dates were essentially converted to thermal time and sum of the degree days above a threshold during a given time period (or sum of the days within a given temperature interval during the period) were calculated. We assumed the lower thermal threshold to be 0°C for wheat and 5°C for aphids. The latter was the common value established using combined data of aphid species [25] and recommended for cereal aphids [26]. We computed DD5 as the sum of daily temperatures above 5°C, and DD05 as the sum of daily temperatures within the [0,5] °C interval.

### Analyses

In order to assess the influence of temperature on the annual variation of aphid population size, we used Generalized Additive Model (GAM) framework assuming normal distribution of errors [27] as the relationship between the response and explanatory variables was inherently non-linear. To this end, we developed four models whose parameters were estimated via Iteratively Reweighted Least Squares (IRWLS) algorithm [27]. In all models we used DD5 and DD05 (see below) as explanatory variables. Additionally, the previous year maximum density or preceding year TSM were used. These alternatives thus differ in the correction for the internal aphid dynamics. As it is not apriori clear which of them is better, we fitted both models and let the data decide which results are better by means of Akaike information criterion (AIC) [28].

The formula of the model (1) correcting for the previous year maximum density is:
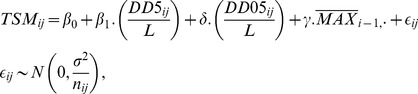
(1)where:

- *TSM_ij_* is TSM for year *i* and plot *j*.

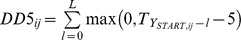
 is the sum of daily temperatures above 5°C within the time period 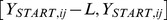, i.e. *L* days back into the past before the aphid season started locally. 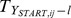 is the average daily temperature at day 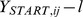. *L* is unknown and has to be estimated from the data (see below). For numerical reasons and also for making comparisons easy, we use standardized version of the DD5 as an explanatory variable in the model (1), namely DD5*_ij_*/*L*.

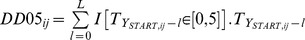
 is the sum of daily temperatures within the [0,5]°C interval. *I*[*condition*] is an indicator function assuming the value of 1 if the condition in its argument is satisfied and 0 otherwise. For numerical reasons and also for making comparisons easy, we use standardized version of the *DD*05 as an explanatory variable, namely *DD*05*_ij_*/*L*.

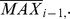
 is the previous year maximum density (averaged over all the plots investigated in year *i*−1).
- *n_ij_* is the number of tillers inspected on plot *j* at time *Y_ij_*.

The model postulates that, conditionally on the covariates, the (true) residuals *ε_ij_* are independently and normally distributed with mean *μ* and variance *σ* ^2^. They are heteroscedastic as the variance is related to the investigation effort *n_ij_* (which is heterogeneous in both space and time).

This regression model allowed estimation of the magnitude of the historical temperature effects on the TSM, separating contributions of DD5 and DD05. By estimating the length of the period over which the DD5 and DD05 are integrated (*L*), the model (1) investigates duration of the past, which is the most relevant from temperature point of view. Quite advantageous is the fact that the estimation of *L* proceeds in a fully empirical way (without apriori knowledge, models or restrictions on *L* are assumed). Furthermore, the model allows for competition between the two temperature-related terms and a term describing the influence of the previous year maximum density. Finally, the model copes with the problem of different amount of information available from different years and plots (as the number of inspected tillers varied) by assuming heteroscedasticity of the random error term. The estimated coefficients can be easily compared against what is expected from biological considerations. For instance, we expect both *β*_1_<0 and *δ*<0, but *γ*≥0 (the effect of past abundance should be positive, if any).

The formula of the model (2) correcting for the previous year time when maximum density was observed is:
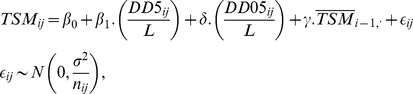
(2)where 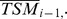 is the previous year TSM (averaged over all the plots investigated in year *i*−1). Other terms and assumptions are the same as in model (1). This model makes a different correction to the nuisance-like influence of the past aphids dynamics.

Both models (1) and (2) were based on traditional degree-days (DD) concept [29] in order to investigate possibly complex and non-homogeneous temperature influence. This concept assumes that e.g. the temperature increase of 1 degree influences the aphids in exactly the same way whether it comes one day before the start of the season or 1 month before the season start. In other words, DD approach assumes time-homogeneous temperature influence (i.e. constant, time-independent *β*_1_ coefficient in both (1) and (2)). That might not be a correct assumption, hence, it was verified from the data via estimating the coefficient without the homogeneity restriction. This was done by allowing *β*_1*l*_ to be different for different lags (*l*). In such model, the effect of temperature increase would be different in different periods (depending on the time before the current aphid season started). The formula of the model with time-inhomogeneous temperature effect was:
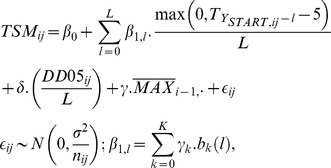
(3)where *β*_1*l*_ is now lag-varying, allowing different temperature influence at days located in differently distant past before the start of the current aphid season. For identifiability reasons, the *β*_1*l*_ coefficients cannot be totally free, but were restricted to lie on the linear combination of *K*+1 basis functions *b_k_*(*l*), for *k* = 0, …, *K*. Specifically, we took B-spline basis and *K* = 9. Such an approach is a slight generalization of the Almon model [30]. Other terms are as in our former models (1) and (2).

Analogously to model (2), which corrects for previous year time when maximum density was observed, the formula with time-inhomogeneous temperature effect is:
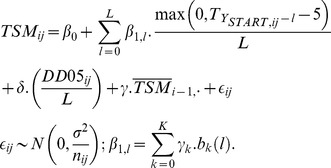
(4)


Main purpose of expanding the model to (3) and (4) is to conduct a sensitivity analysis with respect to the (possible) non-homogeneous temperature effects. To this end, results of models (1) and (3), respectively (2) and (4), are to be compared (i.e. those with constant and non-constant *β*_1_ coefficients).

Additional bonus of this approach is that estimated *β*_1*l*_ coefficients can be plotted as a function of lag (*l*), together with their pointwise confidence intervals. This will allow us to explore the complex dependency of TSM on temperature (the relationship is functional in fact) in a novel way. New information both from practical and theoretical points of view can be obtained by investigating the region(s) (i.e. sets of lags) where *β*_1*l*_ coefficients are significantly different from zero, where they change magnitude fast, etc.

Since the data were obtained from several stands in the same year we checked the effect of possible interdependencies among plots upon the estimated coefficients (most importantly upon their signs and significance). We expanded the model (1) by including random year effects which impose exchangeable correlation among the stands investigated in the same year. Results of this sensitivity analysis showed that the coefficients and their standard errors changed (most of the change was related to decreased precision of the DD05 effect and increased precision of the previous year maximum effect), their structure in terms of the signs and significance, however, remained the same. Parameter estimates from expanded version of the model (1) are available at http://www.vurv.cz/sites/file/cerealaphids.xls. In Results we only present models without random-year effects.

As the counts of aphids were made on two fields during analysis we also tested whether there are no substantial differences between fields. We fitted an extension of the model (1) to the data and included interactions of field with all other effects. None of the interaction effects was significant (p-values>0.50), therefore we combined data from the two fields together.

In all four models the depth of the history, *L*, over which the major temperature influence the dynamic, must have been estimated. Expert knowledge on this estimate was not available so it was estimated from the data. This approach was preferred also because the effect of *L* is not orthogonal on other parameters of interest and hence wrong value of *L* can easily influence (spoil) other parameters. Instead, the value of *L* was inferred from the available data. This is indeed possible via profiled optimization [31]. For the *L* selection, we used AIC and searched for such values (across non-negative integers) where the AIC was minimal.

All the computations were done in R environment [32], with the help of the mgcv package [27].

## Results

**Figure 1:**
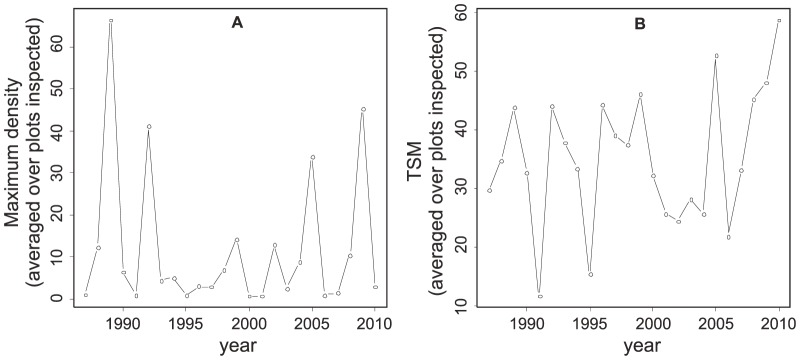
Change in (a) the maximum aphid densities and (b) time elapsed from the start of aphid presence to their maximum abundance on wheat plots (TSM) over 24 years of study.

Population number of aphids showed fluctuating dynamic. Maximum abundances of aphids varied among years with a period of 4 years, approximately. Average values oscillated between 1 (1991) and 67 (1989) (Fig. 1A). Similarly *TSM* varied among years showing similar fluctuating dynamic (Fig. 1B).

**Figure 2:**
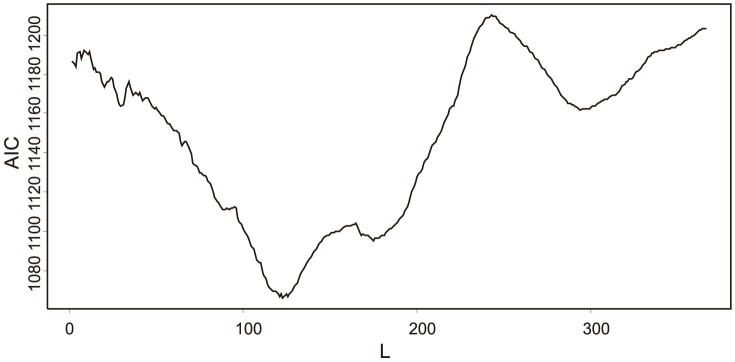
Relationship between the AIC values and *L* using model (1). The curve is a loess fit.

To estimate optimal value of *L*, AIC was plotted on all possible lags for model (1) (Fig. 2). The minimum was attained at *L* = 123 (i.e. 123 days since the beginning of the year). This means that the temperature effect on the *TSM* increases up to lag 123 before the start of the current season. Since the average start of the aphid season was 143 days since the beginning of the year, the temperature influence tends to show its effect, on average, in the second half of January. The optimization of *L* using the model (2) gave identical result. Therefore we used *L* = 123 in all subsequent analyses. Obviously, this means that all subsequent analyses have to be taken as conditional upon the value of *L* = 123. One would need much more data for assessment of the variability induced by the fact that *L* was estimated.

Estimates of all parameters in the model (1), except for *γ*, the density-dependence in terms of the previous year maximum, were significantly different from 0 (Table 1). The model fitted the data quite well (R^2^_adj._ = 0.625). The signs of *β*_1_ and *δ* are perfectly in agreement with our expectation: negative *β*_1_ shows that increasing DD5 leads to decreasing mean of TSM; negative *δ* indicates that increasing DD05 leads to decreasing mean of TSM. The large value of *δ* shows strong effect of DD5 suggesting that in the interval [0,5] °C the wheat is escaping from aphids attacks. The value of *γ* is also in agreement with our expectation. Correlations among non-intercept parameter estimates were not substantial suggesting that the model (1) separates the influence of the explanatory variables quite well.

**Table 1:** Parameter estimates (excluding intercept), their SE and associated p-values from the model (1).

Coefficient (variable)	Estimate	Standard error	p-value
*β*_1_ (*DD5*)	−15.39	1.00	<0.0001
*δ* (*DD05*)	−20.58	3.67	<0.0001
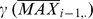	0.08	0.06	0.22

Estimates of the model (2), though based on a different density-dependence term (the previous year TSM), are very similar to those of model (1) (Table 2). This model showed also acceptable fit (R^2^_adj._ = 0.626). Interpretability of the negative sign of *γ* is questionable, but the estimate is not significantly different from 0. Comparison of the quality of the models (1) and (2) in terms of AIC reveals that the models are quite similar (1066.50 vs. 1066.65).

**Table 2:** Parameter estimates (excluding intercept), their SE and associated p-values from the model (2).

Coefficient (variable)	Estimate	Standard error	p-value
*β*_1_ (*DD5*)	−15.88	1.06	<0.0001
*δ* (*DD05*)	−22.65	3.57	<0.0001
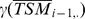	−0.12	0.11	0.24

Structure of the model (3), which allows for lag-varying *β*_1_ coefficient, is similar to the previous models (Table 3). The estimates of *δ* and *γ* are very similar to the estimates of previous models. Very low p-value for *β*_1_ shows that the null hypothesis is rejected: *β*_10_ = *β*_11_ = … = *β*_1*L*_ = 0. Based on AIC, the model (3) is better than model (1) (AIC = 1012.53).

**Table 3:** Parameter estimates (excluding intercept), their SE and associated p-values from the model (3).

Coefficient (variable)	Estimate	Standard error	p-value
*β*_10_, *β*_11_, …, *β*_1*L*_ (*DD5*)	Plotted in Fig. 3	Plotted in Fig. 3	<0.0001 for the overall DD5 effect
*δ* (*DD05*)	−21.16	6.48	0.0014
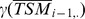	0.08	0.06	0.13

Finally, we fitted model (4). This model showed results quite similar to the model (3), it had AIC = 1011.18 and comparable R^2^_adj_ value (0.756).

**Figure 3:**
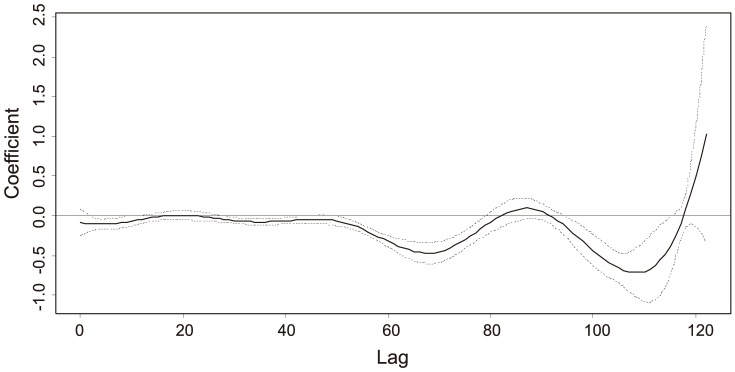
B-spline *fit of estimated β*_1*l*_ coefficient for model (3) as a function of lag *l* with 95% (pointwise constructed) asymptotic confidence interval.

By plotting estimated *β*_1*l*_ coefficients of the model (3) as a function of lag *l* (Fig. 3) we can determine how the estimates are changing with the lag. The temperature influence was far from being homogeneous in time. For most of lags negligible or zero *β*_1*l*_ coefficients were estimated. There are two intervals with distinctly negative *β*_1*l*_ values: roughly 50 to 80 and 90 to 110 days before the beginning of the current aphid season. The positive *β*_1*l*_ estimates for the lags 120 and higher are probably meaningless because of the wide confidence band that includes zero. Quite interesting might be slightly negative estimates near to the season beginning (lags smaller than about 2 weeks). Plot of *β*_1*l*_ *versus* lag *l* obtained from model (4) was quite similar to that from model (3) (thus not shown here).

## Discussion

Our analysis of a long time-series of aphid abundance on winter wheat revealed that annual variation in aphid numbers is related to temperature and could be predicted from temperature data that precede the aphid population growth. The effect of temperature could be expected because temperature determines rate of all processes in ectoterms. The number of aphids essentially depends on length of convenient host plant phase. Thus in years where immigration occurs relatively early to plant physiological age the length of aphid presence increases and aphid populations grow to greater numbers. This decoupling of host plant and aphid development is enabled by differences in their thermal requirements. Obtained results revealed periods in host plant and aphid seasonal cycle that are particularly sensitive to thermal effects. Their biological meaning could be inferred from well established knowledge of physiology and development of wheat and aphids.

### Wheat development

Growth and development of winter wheat is determined by a complex array of environmental and intrinsic factors. No single environmental factor can be used to completely predict development but temperature, photoperiod and intrinsic differences among cultivars are clearly the most critical factors [22]. In our analysis the temperature effect on the period of aphid population growth increased up to 123 days before the start of the current season, i.e. up to the second half of January. This period is apparently related to completion or attaining a particular stage of vernalization. It is a process of thermal induction in which later growth and flowering are promoted by exposure to low temperatures. In winter wheat vernalization may take place in a wide range of temperature conditions between −1.7 and 15.7°C with an optimum of 4.9°C [33]. Chilling is not important for control of heading time but for adaptation to winter coldness [34]. The time required to complete vernalization is affected by temperature fluctuation and also by seedling development stage (timing of autumn sowing) and photoperiod. The local agronomy practice revealed 50–60 d long vernalization period [35] which points to the period indicated by our analysis as a time of completing vernalization requirements.

Increased sum of daily temperatures above 5°C and the sum of daily temperatures within the [0,5] °C interval both shortened the length of period aphid population growth. Significant effect of the latter suggests that in the interval [0,5] °C the wheat is escaping from aphids attacks. Thermal time to full vernalization, the photoperiod at the time of full vernalization, temperature after vernalization and genetically determined narrow-sense earliness of the cultivars affect number of main shoot leaves before transition to flowering and increase the length of the period before heading [36], [37].

### Aphid populations

The strongest temperature effect was about 50 to 80 and 90 to 110 days before the beginning of the current aphid season, i.e. mid of February and March. Apparently both periods are related to particular episodes of aphid seasonal cycle. Typical features of cereal-aphid life history in central Europe should be considered to show biological meaning of these critical periods. The characters important for interpretation of the two critical periods are shared by all species considered in our analysis.

Aphid species have a capacity to overwinter as anholocyclic or holocyclic clones. With anholocyclic overwintering strategy the parthenogenetic aphids colonize autumn sown cereals, more the early sown than late sown stands, and continue to survive in this secondary host plant until spring [38], [39]. In oceanic and Mediterranean climate anholocyclic clones occur exclusively or dominate [40]. Under continental climate winter mortality becomes high and survival is limited to mild winters only [26], [41]. This is due to poor tolerance of virginoparae aphids to low temperatures [26], [42]. As a consequence, holocyclic clones become dominant in continental climate of the Central and Eastern Europe [43]. The species in the autumn produce sexual morphs, overwinter in egg stage on primary host plants and migrate to wheat stands in the spring. In Bohemia the reproduction is nearly exclusively holocyclic. Over a period of 37 years (1976–2013) where aphid populations were surveyed on cereals by different methods, only one probably anholocyclic population was observed (A. Honek, unpubl.).

Because of holocyclic reproduction, the periods of temperature sensitivity established in our analysis address egg development. The eggs laid in late autumn are dormant. Termination of dormancy requires a period of chilling which is 70–100 d in *S. avenae* kept at 7°C [44]. The dormancy is longer in holocyclic clones present in the central Europe than in clones with mixed strategy occurring in oceanic climates [45]. Consequently, we may interpret the period of early February as the time of termination of egg dormancy. Post-dormancy development is then controlled by temperature. It requires a certain sum of effective temperatures. It is not known for cereal aphids but in species with non-diapausing eggs it is about 100 degree days [46], [47]. Hatching occurs once thermal requirements have been completed, which points to March. This coincides with the second period indicated by our analysis. In fact, in the central Europe this hatching time was confirmed by direct observation [48], [49]. About 2–3 generations develop then on primary hosts consisting of an increasing proportion of winged migrants which move to cereal crop stands. Abundance of flying populations is poorly correlated with final aphid numbers [50], [51].

### Population dynamics

Long term studies of abundance of cereal aphids are few. A 26-year series (1970–1995) of seasonal and annual variation of *S. avenae* abundance in winter wheat established by sweeping with a net [52] revealed similar range of variation of maximum annual abundance that varied within 3 orders of magnitude. Series of suction trap catches up to 20 years long were analysed [53]–[55] but are difficult to compare with our data. Our data revealed that aphids may show cyclic dynamic with a period of 4 years, approximately.

The effect of previous year either in the form of maximum density or TSM on aphid density turned out to be insignificant when temperature correction was taken into account. This is partly in contrast with some earlier results. A significant direct density dependence was established in a long time-series of abundance of *M. dirhodum* (in 16 of 16 series), *R. padi* (10 of 17 series) and *S. avenae* (4 of 16 series) [24].

Our results contribute in predicting annual variation in maximum abundance of aphid populations. Cereal aphids have long been subject to population modelling [56]. The models are either focused on predicting time of migration or on growth of populations settled in cereals. The models of aphid migration contain weather parameters including temperature and precipitation [57]. The models of aphid population growth in cereal stands naturally contain temperature as a proxy of biological time and further parameters including aphid reproduction rate, migration, host plant phenology and effect of natural enemies [58], [59]. The models well predict aphid population development after rectification using field data established at the beginning of the season [60]. One factor limiting the length of aphid presence in wheat stands is spring immigration. It is delayed by low winter temperatures, i.e. the cooler the weather in the winter the later the arrival of cereal aphids [57], [61]. Similar to our results, some studies indicated that maximum aphid abundance increases with length of aphid presence in crop stands [62].

Our results are important in the context of expected effects of climate change on populations of cereal aphids. High temperatures of ≥30°C have fatal behavioral consequences [63], increase physiological developmental time and decrease fecundity and survival [64], even when acting for short periods [65]. However, model simulations indicated that the changes in aphid population dynamics resulting from global warming will be probably not dramatic because parallel change in atmospheric CO_2_ concentration and nitrogen fertilization may compensate for temperature effects on aphid development rate, voltinism, production of alatae and effect of natural enemies [66]–[68]. Results of field experiments simulating increase of ambient temperature are still ambiguous [69]. Our analysis is important because it provides insight into mechanism of temperature effects on population dynamic of aphids before the season starts.

Here we show that annual population dynamic of a complex of aphid species on wheat can be predicted using only temperature data long before the onset of aphid immigration to the crop. Unlike other variables, such as natural enemies or aphid reproductive potential that can not be estimated before the aphid population establishes itself, temperature data has potential for use in prediction of aphid occurrence and planning of crop protection management strategies.
